# Elevated serum interferon γ-induced protein 10 kDa is associated with TAFRO syndrome

**DOI:** 10.1038/srep42316

**Published:** 2017-02-13

**Authors:** Noriko Iwaki, Yuka Gion, Eisei Kondo, Mitsuhiro Kawano, Taro Masunari, Hiroshi Moro, Koji Nikkuni, Kazue Takai, Masao Hagihara, Yuko Hashimoto, Kenji Yokota, Masataka Okamoto, Shinji Nakao, Tadashi Yoshino, Yasuharu Sato

**Affiliations:** 1 Department of Pathology, Okayama University Graduate School of Medicine, Dentistry, and Pharmaceutical Sciences, Okayama, Japan; 2 Hematology/Respiratory Medicine Kanazawa University Faculty of Medicine, Institute of Medical, Pharmaceutical and Health Sciences, Kanazawa, Japan; 3 Department of General Medicine, Okayama University Graduate School of Medicine, Dentistry, and Pharmaceutical Sciences, Okayama, Japan; 4 Division of Rheumatology, Kanazawa University Hospital, Kanazawa, Japan; 5 Department of Hematology, Chugoku Central Hospital, Fukuyama, Japan; 6 Division of Clinical Infection Control and Prevention, Niigata University Graduate School of Medical and Dental Sciences, Niigata, Japan; 7 Division of Hematology, Niigata City General Hospital, Niigata, Japan; 8 Department of Hematology, Eiju General Hospital, Tokyo, Japan; 9 Department of Diagnostic Pathology, Fukushima Medical University School of Medicine, Fukushima, Japan; 10 Division of Pathophysiology, Okayama University Graduate School of Health Sciences, Okayama, Japan; 11 Department of Hematology and Medical Oncology, Fujita Health University School of Medicine, Toyoake, Japan

## Abstract

Multicentric Castleman disease (MCD) is a heterogeneous lymphoproliferative disorder. It is characterized by inflammatory symptoms, and interleukin (IL)-6 contributes to the disease pathogenesis. Human herpesvirus 8 (HHV-8) often drives hypercytokinemia in MCD, although the etiology of HHV-8-negative MCD is idiopathic (iMCD). A distinct subtype of iMCD that shares a constellation of clinical features including thrombocytopenia (T), anasarca (A), fever (F), reticulin fibrosis (R), and organomegaly (O) has been reported as TAFRO-iMCD, however the differences in cytokine profiles between TAFRO-iMCD and iMCD have not been established. We retrospectively compared levels of serum interferon γ-induced protein 10 kDa (IP-10), platelet-derived growth factor (PDGF)-AA, interleukin (IL)-10, and other cytokines between 11 cases of TAFRO-iMCD, 6 cases of plasma cell type iMCD, and 21 healthy controls. During flare-ups, patients with TAFRO-iMCD had significantly higher serum IP-10 and tended to have lower PDGF-AA levels than the other 2 groups. In addition, serum IL-10, IL-23, and vascular endothelial growth factor-A were elevated in both TAFRO-iMCD and iMCD. Elevated serum IP-10 is associated with inflammatory diseases including infectious diseases. There was a strong correlation between high serum IP-10 and the presence of TAFRO-iMCD, suggesting that IP-10 might be involved in the pathogenesis of TAFRO-iMCD.

Multicentric Castleman disease (MCD) is a rare inflammatory disorder often characterized by inflammatory flare-ups including episodes of systemic inflammation. It may be characterized by fever, cachexia, systemic lymphadenopathy, polyclonal hypergammaglobulinemia, microcytic anemia, hypoalbuminemia, and elevated serum inflammatory proteins, such as C-reactive protein (CRP)^1^,^2^,^3^. Many clinical manifestations of MCD are associated with overexpression of interleukin (IL)-6^4^,^5^.

Clinical studies have shown that human herpesvirus (HHV)-8, which infects B-cells and expresses a viral homolog of IL-6, drives the disease in HHV-8-infected MCD (HHV-8-associated MCD), especially in immunocompromised patients^6^,^7^. However, HHV-8-negative MCD or idiopathic MCD (iMCD) has been observed in a significant number of patients with MCD^8^,^9^.

In a previous prospective study, we reported the clinicopathological analysis of 25 cases of a unique subtype of iMCD characterized symptomatically by thrombocytopenia (T), anasarca (A), fever (F), reticulin fibrosis (R), and organomegaly (O), and known as TAFRO syndrome (TAFRO-iMCD)^10^. Patients with TAFRO-iMCD were also shown to have elevated serum interleukin (IL)-6^10^,^11^,^12^. Because IL-6 is a proinflammatory cytokine that stimulates B-cell maturation, increases immunoglobulin production by plasma cells, and stimulates megakaryocytes, elevations in IL-6 typically cause thrombocytosis and polyclonal hypergammaglobulinemia^13^,^14^,^15^,^16^. However, patients with TAFRO-iMCD exhibit thrombocytopenia without polyclonal hypergammaglobulinemia^10^,^11^,^12^. These findings suggest that elevated serum IL-6 might not be a primary pathological driver of the proinflammatory hypercytokinemia observed in patients with TAFRO-iMCD.

Therefore, in the present study, to identify the cytokine profile associated with TAFRO-iMCD, we compared serum cytokine profiles between patients with non-HHV-8-associated MCD, including TAFRO-iMCD and plasma cell iMCD (iMCD-NOS), and healthy subjects.

## Results

### Clinical features

The patients’ clinical and laboratory features are summarized in Table 1. Myelofibrosis (MF) was scored using a scale from 0 to 3 according to the European Consensus on grading bone marrow fibrosis^17^. In the TAFRO-iMCD group, the 6 cases that were positive for reticulin fibrosis were classified as MF-1 and had a very loose network of reticulin fibers. Bone marrow samples were not available for the iMCD-NOS cases; therefore, the status of reticulin fibrosis could not be evaluated.

### Laboratory features of TAFRO-iMCD and iMCD-NOS

Detailed laboratory data are presented in Table 1. Patients with TAFRO-iMCD and iMCD-NOS commonly demonstrated microcytic anemia, hypoalbuminemia, and elevated serum CRP. Serum procalcitonin (PCT) levels for all 3 cases of TAFRO-iMCD were high (median, 2.0 ng/mL; range, 1.4–15.68 ng/mL). Compared with the iMCD-NOS group, patients in the TAFRO-iMCD group had severe thrombocytopenia and elevated serum ALP without polyclonal hypergammaglobulinemia.

### Cytokine profiles during flare-ups

**Figure 1:**
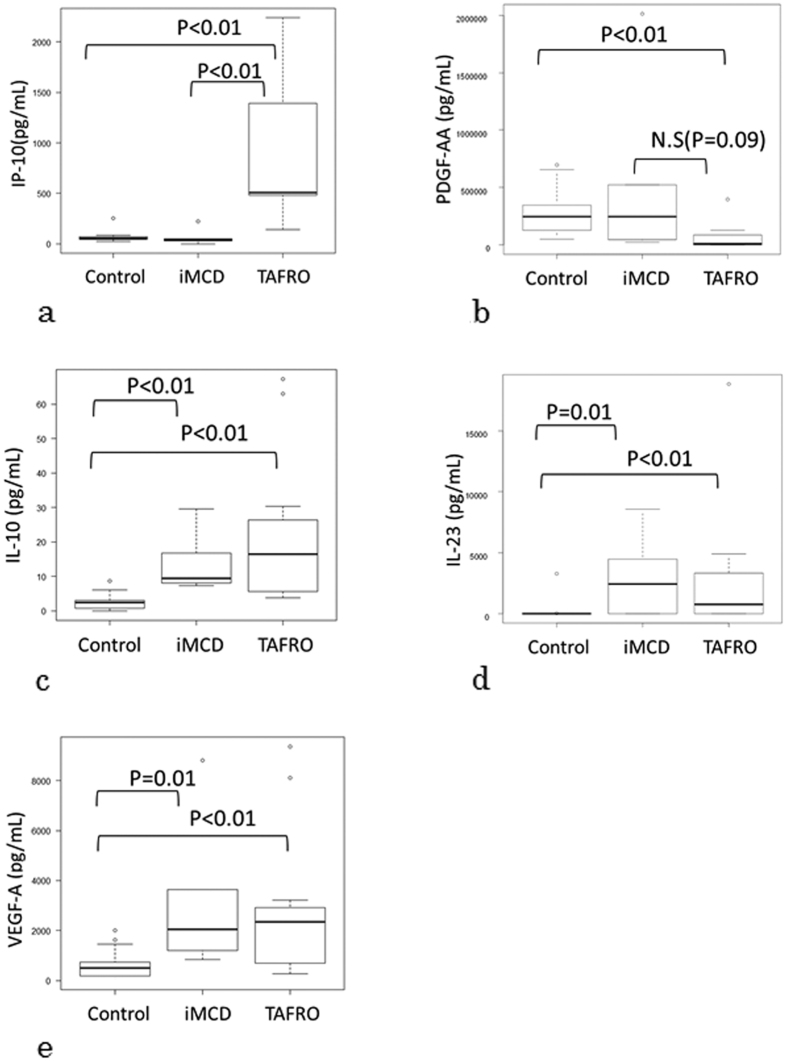
Steel-Dwass test analysis of between-group differences. (**a**) The median serum IP-10 level was significantly higher in the TAFRO-iMCD group than in the other 2 groups. (**b**) The median serum PDGF-AA level was significantly lower in the TAFRO-iMCD group than in controls and tended to be lower in the TAFRO-iMCD groups than in the iMCD-NOS group. The serum IL-10 (**c**), IL-23 (**d**), and VEGF-A (**e**) levels were significantly higher in the TAFRO-iMCD and iMCD-NOS groups than in the control group. Control: Healthy control, iMCD: iMCD-NOS, TAFRO: TAFRO-iMCD, IP-10: chemokine interferon γ-induced protein 10 kDa, PDGF-AA: platelet-derived growth factor -AA, IL: interleukin, VEGF-A; vascular endothelial growth factor-A, N.S: not significant.

The cytokine levels in the 3 groups are presented in Table 2. When significant between-group differences were detected, the data were analyzed using the Steel-Dwass test (Fig. 1). The median serum IP-10 level was significantly higher in the TAFRO-iMCD group than in the other 2 groups (P < 0.01; Fig. 1a). The median serum PDGF-AA level was significantly lower in the TAFRO-iMCD group than in controls (P < 0.01; Fig. 1b) and tended to be lower in the TAFRO-iMCD group than in the iMCD-NOS group (P = 0.09; Fig. 1b). Serum IL-10 (P < 0.01), IL-23 (P < 0.01), and VEGF-A (P < 0.01) levels were significantly higher in the TAFRO-iMCD and iMCD-NOS groups than in the control group (Fig. 1c–e).

The serum IL-13 levels tended to be higher in the iMCD-NOS group than in the controls (P = 0.05) and tended to be lower in the TAFRO-iMCD group than in the controls (P = 0.06) (data not shown). The serum IL-1β, IL-5, IL-27, and IL-12p70 tended to be higher in the iMCD-NOS group than in the other 2 groups (Table 2).

The serum IFN-γ, IL-17A, IL-2, IL-9, IL-22, IL-6, IL-4, and TNF-α levels were not significantly different between the 3 groups (Table 2).

## Discussion

Although human and animal studies have demonstrated that IL-6 plays a major role in the symptomatology and pathogenesis of MCD, there are few reports concerning other cytokine profiles in MCD, especially in non-HHV-8-associated MCD. Here, we report the serum cytokine profiles in patients with non-HHV-8-associated MCD including TAFRO-iMCD and iMCD-NOS. This is the first report to evaluate the cytokine profile in patients with TAFRO-iMCD.

Notably, during flare-ups, patients with TAFRO-iMCD had significantly elevated serum IP-10 compared to that in patients with iMCD-NOS or healthy subjects. IP-10 is a cytokine belonging to the CXC chemokine family. Its expression has been associated not only with autoimmune disease, but also with inflammatory diseases including viral, bacterial, and mycotic infectious, immune dysfunction, and tumorigenesis. Many pathogens have been shown to harbor IP-10 including rhinovirus, respiratory syncytial virus, Coxsackie virus, mycoplasma, tuberculosis, and *Candida albicans*. However, the pathogenic mechanism(s) of IP-10 in infectious diseases remains unclear^18^,^19^.

In a previous retrospective case series, we reported that >30% of patients with TAFRO-iMCD had abdominal pain on diagnosis, and some patients who underwent a liver biopsy had neutrophilic infiltration and microabscesses, mostly in the portal region (unpublished data). Although the number of samples was limited, serum PCT levels in patients with TAFRO-iMCD were elevated in this study. High levels of serum PCT are associated with systemic or severe infection, especially bacterial infection, but not viral infection^20^. It is known that IFN-γ plays an important role in early antiviral defense mechanisms, and IFN-γ secretion inhibits PCT induction^21^. The serum IFN-γ levels of patients with TAFRO-iMCD in this study were not elevated. The elevated serum IP-10 levels in patients with TAFRO-iMCD in our study might suggest that this hypercytokine syndrome could be triggered by an undetected infection in the hepatobiliary system. Furthermore, some reports have suggested that the sudden onset of TAFRO-iMCD might also support this hypothesis^10^,^11^,^12^.

PDGF is a major serum mitogen for cells of mesenchymal origin, which has been implicated both directly and indirectly in several pathological states including bone marrow sclerosis, neoplasia, arthritis, and arteriosclerosis^22^,^23^. PDGF is stored in the alpha granules of platelets, and released upon platelet activation during platelet aggregation^24^. Elevated PDGF has been noted to be concomitant with thrombocytosis in diseases such as essential thrombocythemia^25^. Interestingly, it has been reported that PDGF levels were elevated in patients with immune thrombocytopenic purpura (ITP)^26^. ITP is a clinical syndrome characterized by a decrease in circulating platelets due to peripheral thrombocyte consumption caused by autoantibodies against platelet antigens. It also is characterized by an increase in the number, size, and ploidy of bone marrow megakaryocytes. The patients with TAFRO-iMCD in our study also showed marked thrombocytopenia and hypermegakaryocytes, resembling ITP; however, patients with TAFRO-iMCD had much lower serum PDGF-AA levels than those noted in ITP. These findings suggest that thrombocytopenia in TAFRO-iMCD is not caused by increased peripheral thrombocyte consumption alone, but also by megakaryocyte dysfunction.

In human immunodeficiency virus-infected patients, elevated plasma IL-10 is associated with exacerbation of HHV-8-associated MCD^27^,^28^. Although in the present study, both TAFRO-iMCD and iMCD-NOS patients had high serum IL-10 levels, the correlation between cytokine levels and clinical outcomes could not be evaluated because of the small sample size.

In conclusion, TAFRO-iMCD and iMCD-NOS shared some common cytokine profiles, some of which were similar to those observed in HHV-8-associated MCD. Furthermore, TAFRO-iMCD had a distinct cytokine spectrum characterized by high levels of IP-10. These findings might be key for understanding the pathogenesis of this hypercytokine lymphoproliferative disorder.

## Materials and Methods

### Study population

The present study investigated 17 patients, all of Japanese descent, diagnosed with HHV-8-negative iMCD, including 11 patients with TAFRO-iMCD and 6 patients with iMCD-NOS, who were evaluated at the Department of Pathology, Okayama University between 1999 and 2013. We assessed 21 healthy subjects as controls. Clinicopathological data were reviewed retrospectively by pathologists and physicians. Informed consent for the use of their samples in research was obtained from patients. All patients’ samples and medical records (clinical history and treatment) were obtained with the approval of the Institutional Review Board (IRB) at Okayama University. The samples were limited to excess human material; therefore, the IRB waived the need for written consent from the patients.

### TAFRO-iMCD

Patients who had at least 3 of 5 TAFRO clinical symptoms and specific TAFRO-iMCD lymph node histopathology^10^,^29^ were classified as having TAFRO-iMCD. Patients’ clinicopathological features were compatible with the diagnostic criteria for TAFRO-iMCD that we proposed previously^10^ (Table 3). In total, 11 patients met the diagnostic criteria, and their serum samples were preserved by freezing. All serum samples were collected during clinical flare-ups. Serum samples from 7 patients were collected at diagnosis before any anti-inflammatory treatments such as corticosteroids or tocilizumab. Serum samples were collected from the other 4 patients while being treated with anti-inflammatory therapies that failed to control disease activity. Of these patients, 3 were treated with prednisolone and 1 was treated with prednisolone and tocilizumab. All cases had previously been recorded as TAFRO syndrome^10^,^29^,^30^.

### iMCD-NOS

Files from the Pathology Department of Okayama University were reviewed and 6 patients with iMCD-NOS were selected from these. Serum samples collected during clinical flare-ups were preserved by freezing. Serum samples were collected from 3 patients at diagnosis before any anti-inflammatory treatments, and from the other 3 patients during prednisolone treatment which did not control disease activity. All cases were reported previously as TAFRO-iMCD^10^.

### Healthy controls

The controls were 21 healthy subjects (11 males and 10 females, aged 27–70 years with a mean age of 47 years of Japanese descent. They had no history of vaccinations or acute or chronic infections within the previous 6 months. Serum samples from all participants were collected and stored at −80 °C until use.

### Diagnostic criteria and clinical disease

In the absence of a consensus definition for the diagnostic criteria of TAFRO-iMCD, we used the diagnostic criteria for TAFRO-iMCD^10^ that we proposed previously (Table 3). For a positive diagnosis, TAFRO-iMCD must meet the histopathological criteria (3 major criteria and ≥ 1 minor criterion). Exclusion criteria were based on possible differential diagnoses including rheumatic diseases such as systemic lupus erythematosus, infectious diseases, and neoplastic diseases such as lymphoma, POEMS syndrome (polyneuropathy, organomegaly, endocrinopathy, M-protein, and skin pigmentation), and cancer.

Histopathological inclusion criteria were negative latency-associated nuclear antigen-1, indicating a lack of HHV-8 infection, and the presence of atrophic germinal centers with enlarged nuclei in endothelial cells, the proliferation of endothelial venules with enlarged nuclei in the interfollicular zone, and small numbers of mature plasma cells in lymph nodes.

The major inclusion criteria for TAFRO were the presence of 3 of the following 5 characteristics: thrombocytopenia, platelet count <100 × 10^3^/μL; anasarca, the presence of pleural fluids and ascites on computed tomography; fever, a body temperature of >38.0 °C (100.4 °F); reticulin fibrosis, evaluated via bone marrow biopsy; organomegaly, small volume lymphadenopathy, hepatomegaly, or splenomegaly on computed tomography; the absence of hypergammaglobulinemia. Minor inclusion criteria included hyper- or normoplasia of megakaryocytes in the bone marrow and high levels of serum alkaline phosphatase (ALP) without marked elevations of serum transaminases.

There is also no consensus definition of a clinical flare-up of MCD. However, we defined a flare-up as requiring treatment due to the presence of at least 1 clinical symptom (e.g., fever, sweating, cachexia, or anasarca) and 1 laboratory abnormality (e.g., hypoalbuminemia or anemia).

### Measurement of cytokines and chemokines

Serum cytokines IL-1β, IL-2, IL-4, IL-5, IL-6, IL-9, IL-10, IL-12p70, IL-13, IL-17A, IL-22, IL-23, IL-27, interferon (IFN)-γ, tumor necrosis factor (TNF)-α, platelet-derived growth factor (PDGF)-AA, vascular endothelial growth factor (VEGF)-A, and chemokine interferon γ-induced protein 10 kDa (IP-10) were analyzed using a FlowCytomix™ kit (Bender MedSystems, Vienna, Austria) and MACSQuant^®^ Analyzer (Miltenyi Biotec, Bergisch Gladbach, Germany) in accordance with the manufacturers’ instructions. The thresholds of detection were as follows: 1.2 pg/mL for IL-6; 1.5 pg/mL for IL-9 and IL-12p70; 1.6 pg/mL for IFN-γ and IL-5; 1.9 pg/mL for IL-10; 2.5 pg/mL for IL-17A and PDGF-AA; 3.2 pg/mL for TNF-α; 4.2 pg/mL for IL-1β; 4.5 pg/mL for IL-13; 6.0 pg/mL for IP-10; 7.2 pg/mL for VEGF-A; 10 pg/mL for IL-27; 16.4 pg/mL for IL-2; 20.8 pg/mL for IL-4; 21.9 pg/mL for IL-23; and 43.3 pg/mL for IL-22.

### Statistical analyses

All statistical analyses were performed using EZR (Saitama Medical Center, Jichi Medical University, Saitama, Japan), which is a graphical user interface for R (The R Foundation for Statistical Computing, Vienna, Austria)^31^.

We categorized cases into 3 groups as TAFRO-iMCD, iMCD-NOS, and healthy controls. The Kruskal-Wallis test was to compare the cytokine and chemokine levels between the 3 groups. When a between-group difference was detected, the Steel-Dwass test was added. A statistically significant difference between the 2 groups was determined using the nonparametric Mann-Whitney U test. Comparisons of the clinical parameters, including platelet count, serum hemoglobin, CRP, albumin, creatinine, immunoglobulin G, immunoglobulin A, immunoglobulin M, and ALP which were measured in fresh blood samples from TAFRO-iMCD and iMCD-NOS patients, were performed using the Fisher’s exact test for categorical variables and the Mann–Whitney U test for continuous variables. All P-values were two-sided, and a P-value ≤ 0.05 was considered statistically significant.

## Additional Information

**How to cite this article:** Iwaki, N. *et al*. Elevated serum interferon γ-induced protein 10 kDa is associated with TAFRO syndrome. *Sci. Rep.* **7**, 42316; doi: 10.1038/srep42316 (2017).

**Publisher's note:** Springer Nature remains neutral with regard to jurisdictional claims in published maps and institutional affiliations.

## Figures and Tables

**Table 1:** Clinical characteristics and laboratory data for TAFRO-iMCD and iMCD-NOS patients.

		TAFRO-iMCD (n = 11)		iMCD-NOS (n = 6)		P value
		Median	(range)	Median	(range)	
Age (years)		51	(39–66)	39.5	(28–66)	NS
Gender (male:female)		8:3		4:2		NS
Anasarca		100%	(11/11)	0%	(0/5)	P < 0.01
Fever		81.8%	(9/11)	0%	(0/6)	P < 0.01
Lymphadenopathy		100%	(11/11)	100	(6/6)	NS
Reticulin fibrosis		85.7%	(6/7)	ND		NE
Plt	(x10^3^ μL)	41	(14–171)	348	(295–473)	P < 0.01
Hb	(g/dL)	9.5	(6.2–14.0)	10.6	(9.0–12.7)	NS
Alb	(g/dL)	2.1	(1.2–3.5)	3.1	(2.5–3.4)	P < 001
CRP	(mg/dL)	9.97	(1.65–26.52)	5.25	(4.10–12.17)	NS
IgG	(mg/dL)	1,672	(1,208.8–2,611)	5,168	(3,947–6,750)	P < 0.01
IgA	(mg/dL)	221	(142.7–426.6)	617	(420–964)	P < 0.01
IgM	(mg/dL)	71	(37–257)	218	(168–365)	P < 0.01
ALP	(IU/L)	672	(179–2,388)	242.5	(174–310)	P = 0.02
Cr	(mg/dL)	1.08	(0.74–6.08)	0.71	(0.46–2.2)	P = 0.02

Anasarca: pleural fluid and ascites, Fever: >38.0 °C (100.4 °F), Plt: Blood platelet count, Hb: Hemoglobin, Alb: Albumin, CRP: C-reactive protein, IgG: Immunoglobulin G, IgA: Immunoglobulin A, IgM: Immunoglobulin M, ALP: alkaline phosphatase, Cr: creatinine, NS: not significant, NE: not evaluated, ND: no data.

**Table 2:** Serum cytokine profiles and Kruskal-Wallis test between TAFRO-iMCD, iMCD-NOS, and healthy control groups.

(pg/mL)	IP-10	PDGF-AA	IL-10	IL-23	VEGF-A	IL-13
TAFRO-iMCD	507.2 (143.2–2.2 × 10^3^)	9.2 × 10^3^ (306.7–395 × 10^3^)	16.5 (3.8–67.3)	753.9 (0–18834.6)	2357.1 (281.4–9361.7)	54.6 (0–138.5)
iMCD-NOS	38.7 (0–221.9)	245 × 10^3^ (21 × 0^3^–2012 × 10^3^)	9.5 (7.4–29.6)	2432.6 (0–8571.5)	2056.7 (843.1–8801.7)	113.5 (0–164.8)
Healthy controls	54.0 (22.7–254.5)	244 × 10^3^ (50 × 10^3^–698 × 10^3^)	2.4 (0–8.7)	0 (0–3265.1)	501.9 (18.1–2007.5)	68.9 (0–114.8)
	P < 0.01	P < 0.01	P < 0.01	P < 0.01	P < 0.01	P < 0.01
**(pg/mL)**	**IL-5**	**IL-27**	**IL-12p70**	**IL-1β**	**IFN-γ**	**IL-17A**
TAFRO-iMCD	0 (0–42.3)	5.4 (0–112.9)	12.09 (0–20.2)	19.2 (0–84.2)	0 (0–10.6)	0 (0)
iMCD-NOS	25.7 (0–61.1)	19.6 (5.9–1718.3)	15.98 (0–31.0)	85.7 (0–277.6)	0 (0–167.8)	0 (0–1058.7)
Healthy controls	0 (0–55.9)	0 (0–46.0)	12.83 (0–14.5)	36.7 (0–84.2)	0 (0)	0 (0)
	P = 0.03	P = 0.02	P = 0.04	P = 0.05	NS	NS
**(pg/mL)**	**IL-2**	**IL-9**	**IL-22**	**IL-6**	**IL-4**	**TNF-α**
TAFRO-iMCD	39.2 (11.8–83.9)	0 (0–20.8)	0 (0–491.6)	0 (0–130.7)	0 (0–14.1)	14.0 (9.3–387.9)
iMCD-NOS	75.2 (0–396.0)	2.2 (0–814.4)	270.8 (0–758.1)	0 (0–13.8)	0 (0–102.2)	30.9 (10.9–61.6)
Healthy controls	35.7 (0–191.2)	0 (0–228.6)	160.8 (0–880.1)	0 (0)	0 (0–14.6)	15.7 (7.9–28.8)
	NS	NS	NS	NS	NS	NS

IP-10: chemokine interferon γ-induced protein 10 kDa, PDGF-AA: platelet-derived growth factor-AA, IL: interleukin, VEGF-A; vascular endothelial growth factor-A, IFN-γ: interferon-γ, TNF-α: tumor necrosis factor- α, NS: not significant.

**Table 3:** Proposed diagnostic criteria for TAFRO-iMCD.

1. Histopathological Criteria;
• Compatible with pathological findings of lymph nodes as TAFRO-iMCD*
• Negative LANA-1 for HHV-8
2. Major criteria;
• Presents 3 of 5 TAFRO symptoms at time of diagnosis **
√ Thrombocytopenia
√ Anasarca
√ Fever
√ Reticulin fibrosis
√ Organomegaly
• Absence of hypergammaglobulinemia
• Small volume lymphadenopathy
3. Minor criteria need 1 or more;
• Hyper/normoplasia of megakaryocytes in bone marrow
• High levels of serum ALP without marked elevated serum transaminase ***

Requirements; fulfilled histopathological criteria, major criteria, and meets 1 or more of minor criteria.

Differential diagnosis is necessary from other disorders, including rheumatologic disease; systemic lupus erythematosus (SLE), neoplastic disease; lymphoma, POEMS syndrome, and cancer.

*TAFRO characteristic findings of lymph node, ie, atrophic germinal centers with enlarged nuclei of endothelial cells, proliferation of endothelial venules with enlarged nuclear in interfollicular zone, and small numbers of mature plasma cells. **Thrombocytopenia: Platelet count < 100 × 103/μ L. Anasarca: the presence of pleural fluids and ascites on computed tomography, Fever: The body temperature of > 38.0 °C (100.4 °F). Reticulin fibrosis: evaluated via bone marrow biopsy, Organomegaly: including lymphadenopathy, hepatomegaly or splenomegaly on computed tomography. ***ALP: alkaline phosphatase.
